# Human Pulp Response to Direct Pulp Capping and Miniature Pulpotomy with MTA after Application of Topical Dexamethasone: A Randomized Clinical Trial

**DOI:** 10.7508/iej.2016.02.002

**Published:** 2016-03-20

**Authors:** Seyed Amir Mousavi, Jamileh Ghoddusi, Nooshin Mohtasham, Shirin Shahnaseri, Payam Paymanpour, Jun-Ichiro Kinoshita

**Affiliations:** a *Torabinejad Dental Research Center, Department of Endodontics, Dental School, Isfahan University of Medical Sciences, Isfahan, Iran;*; b *Dental Research Center, Dental School, Mashhad University of Medical Sciences, Mashhad, Iran; *; c *Department of Oral Pathology, Dental School, Mashhad University of Medical Sciences, Mashhad, Iran;*; d *Dental Implant Research Center, Department of Oral and Maxillofacial Surgery, Dental School, Isfahan University of Medical Sciences, Isfahan, Iran;*; e *Department of Endodontics, Dental School, Shahid Beheshti University of Medical Sciences, Tehran, Iran;*; f *Department of Conservative Dentistry, Showa University Dental Hospital, Tokyo, Japan*

**Keywords:** Dexamethasone, Direct Pulp Capping, Mineral Trioxide Aggregate, Miniature Pulpotomy, Vital Pulp Therapy

## Abstract

**Introduction:**

The aim of this randomized clinical trial was to compare the histologic pulp tissue response to one-step direct pulp capping (DPC) and miniature pulpotomy (MP) with mineral trioxide aggregate (MTA) after application of dexamethasone in healthy human premolars.

**Methods and Materials:**

Forty intact premolars from 10 orthodontic patients, were randomly chosen for DPC (*n*=20) or MP (*n*=20). In 10 teeth from each group, after exposure of the buccal pulp horn, topical dexamethasone was applied over the pulp. In all teeth the exposed/miniaturely resected pulp tissue was covered with MTA and cavities were restored with glass ionomer. Teeth vitality was evaluated during the next 7, 21, 42, and 60 days. Signs and/or symptoms of irreversible pulpitis or pulp necrosis were considered as failure. According to the orthodontic schedule, after 60 days the teeth were extracted and submitted for histological examination. The Kruskal-Wallis and Fisher’s exact tests were used for statistical analysis of the data (*P*=0.05).

**Results:**

Although dexamethasone specimens showed less inflammation, calcified bridge, pulpal blood vasculature, collagen fibers and granulation tissue formation were not significantly different between the groups (*P*>0.05).

**Conclusion:**

Topical dexamethasone did not hindered pulp healing but reduced the amount of underlying pulpal tissue inflammation after DPC and MP in healthy human premolars.

## Introduction

Owing to defense reactions and reparative procedures of pulp-dentin complex to noxious stimuli, preservation of the vitality of this complex is absolutely critical for the survival of a tooth [1, 2]. The aim of different vital pulp therapy (VPT) techniques including direct pulp capping (DPC), is to seal the pulp wound, induce odontoblast-like cell differentiation, and stimulate dentin secretion and mineralization [3]. In 2012, Asgary and Ahmadyar [4, 5] hypothesized that miniature pulpotomy (MP) procedure will result in improved treatment outcome compared to DPC. 

In MP procedure, the infected dentin chips and damaged pulp tissue, especially the injured odontoblast cell layer, will be removed following caries exposure which is more confident in comparison with DPC [6].

Pulp tissue capping with biocompatible/bioinductor materials can stimulate the formation of mineralized tissues at the exposure site [7]. Several different capping materials to date were introduced meeting certain objectives of VPT. Mineral trioxide aggregate (MTA) has been proposed as material of choice for DPC procedures instead of calcium hydroxide because of its clinically easier use, less pulpal inflammation, and more predictable hard tissue barrier formation [8, 9]. Success of Degeneration or necrosis of dental pulp may be a direct consequence of chronic inflammation in the underlying pulpal tissue. Since the first application of topical steroids in 1958 [12], various research studies investigated possible effects of corticosteroids application on dental pulp tissue [13-18]. Although relief of symptoms and continued positive response to vitality tests in chronically inflamed pulps were shown for a steroid containing capping agent, Ledermix (Ledermix; Lederle Pharmaceuticals, Wolfratshausen, Germany) [19]. However, uncertain resolution of pulpal inflammation [20] and unpredictable results of this agent were reported [21, 22]. 

**Figure 1:**
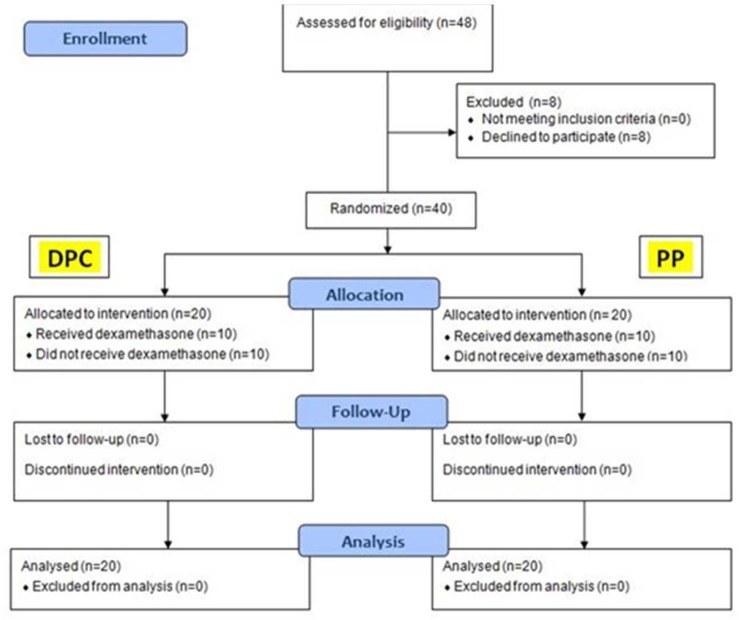
The CONSORT flow diagram of the study

VPT in management of cariously exposed vital permanent teeth has been shown [10, 11].

The anti-inflammatory effect of corticosteroids is well-known but cannot be tested in a cell culture systems. Direct application of dexamethasone to the pulpal tissues may affect the underlying tissue inflammation and reparative events leading to tissue healing. 

To our knowledge, no clinical study has been published about probable effects of direct application of dexamethasone to human dental pulp during DPC and/or MP procedures. Thus the purpose of this prospective randomized clinical trial was to compare the histologic pulp tissue response following DPC and MP using MTA with or without application of dexamethasone in healthy human premolars.

## Materials and Methods

This randomized, double-blind, placebo-controlled, parallel-group study was conducted with volunteers recruited from the pool of orthodontic patients at Mashhad Dental School. The study protocol was approved by the Mashhad University of Medical Sciences Ethical Committee and was registered at ClinicalTrials.gov (Identifier: NCT02574468).

All healthy adult patients who had four intact fully developed maxillary and mandibular premolars needed to be extracted because of orthodontic treatment plan were considered eligible. Exclusion criteria were history of previous trauma, clinical and/or radiographic caries, restorations, periodontal problems, intake of anti-inflammatory medication before (over the last couple of weeks) and during the study, and known allergy and/or contraindications to corticosteroids. Of the 12 confirmed patients (48 teeth), 2 patients (8 teeth) refused to take part in the study. A total of 40 teeth from 10 patients (18-30 year old patients with an average of 24 years) were included in this clinical trial and randomly assigned to the test groups (Figure 1). After giving thorough and careful explanation of the study, verbal and written informed consent was acquired from all patients or their parents.

***Clinical procedures***

Using simple randomization procedures (computerized random numbers), forty intact premolars were randomly assigned to each of 4 treatment groups (*n*=10) including DPC, MP, DPC+dexamethasone and MP+dexamethasone. 

After administration of local anesthesia with 3% plain mepivacaine (Septodont, Cedex, France), rubber dam was applied and tooth surface was disinfected with 2% chlorhexidine gluconate. Occlusal cavity was prepared using high speed diamond fissure bur and buccal pulp horn was mechanically exposed (approximately 1 mm in diameter) using a sterile high speed carbide round bur. In MP groups, the depth of penetration to the pulp was 2 mm. All of the cavity preparation procedures were intermittently performed under copious water coolant and light hand pressure. Using a sterile 5.25% NaOCl-soaked cotton pellet, bleeding from the exposure site was controlled and the cavity was rinsed with 2 mL of sterile saline. 

**Figure 2:**
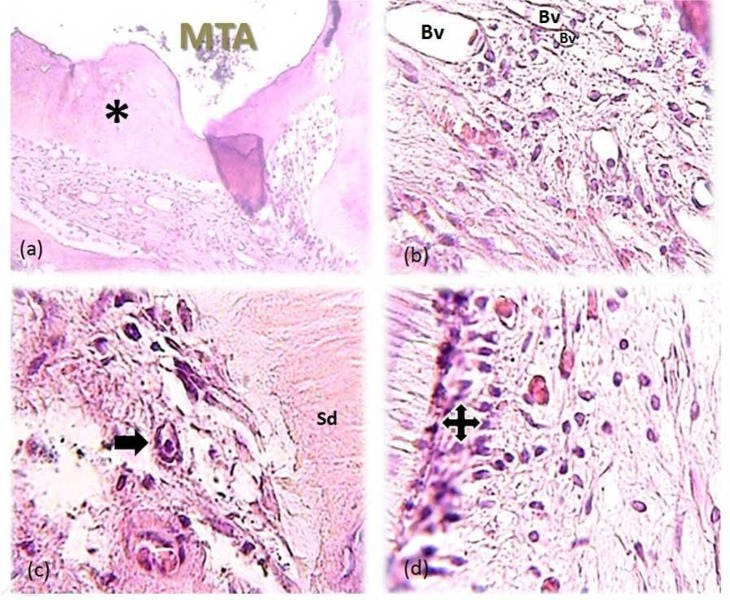
*A)* Complete calcified bridge formed (asterisk) following DPC (100×); *B)* A 400× view of the same tooth, note the presence of macrophages (arrow) as a reaction to the capping material around the blood vessels; *C)* Secondary dentin (SD); *D)* Odontoblast-like cells in odontoblastic layer (quad arrow

**Figure 3:**
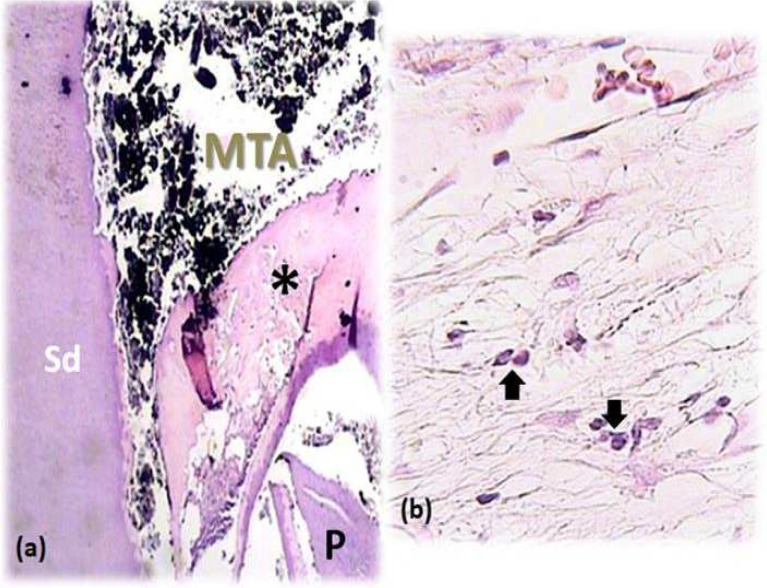
*A) *Incomplete calcified bridge formation (asterisk) after DPC. Note the pulp (P) and secondary dentin (SD) (40×); *B*) Presence of Macrophages and histocytes (arrows) reflects tissue reaction to the capping material (400×)

For blinding the practitioner to the materials, dexamethasone (Sigma, La Verpillere, France) and distilled water were held in identical light proof syringes and numbered for each tooth according to the randomization schedule. The concentration/dose of dexamethasone applied was 10^-8 ^M. Each tooth was assigned an order number and its pulpal wound received the solution in the corresponding prepacked syringe prior to sealing off the exposure site with white ProRoot MTA (Dentsply, Tulsa Dental, Tulsa, OK, USA); MTA was mixed according to the manufacturer's directions and a wet cotton pellet was placed over the material for 10 min. Eventually, the coronal cavity was restored with light-cured resin modified glass ionomer (Fuji II LC; GC Corporation, Tokyo, Japan) and cured for 40 sec using a curing light. Pulp and periapical status were clinically (7, 21, 42, and 60 days) and radiographically evaluated (42, and 60 days) during the study. Clinical and radiographic signs and/or symptoms of irreversible pulpitis or pulp necrosis were considered as treatment failure. After sixty days, teeth were extracted atraumatically and prepared for submission to the oral pathology department.

**Figure 4:**
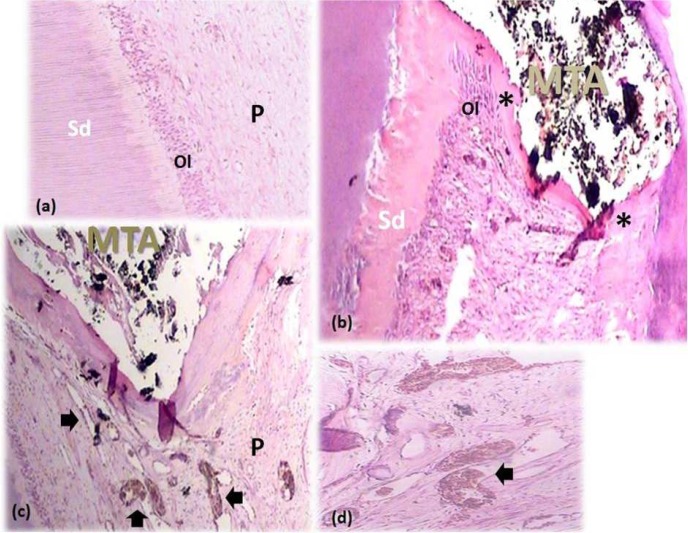
*A) *Normal odontoblastic layer (Ol) and unchanged collagen fibers. Note the pulp (P) and secondary dentin (SD) (100×); *B *and* C*) Complete calcified bridge formation (asterisks) following MP. Note the increased vascularity (arrows). (40×); *D*) A 100× view of the same tooth represents the increased pulpal vascularity under the capping material

**Figure 5:**
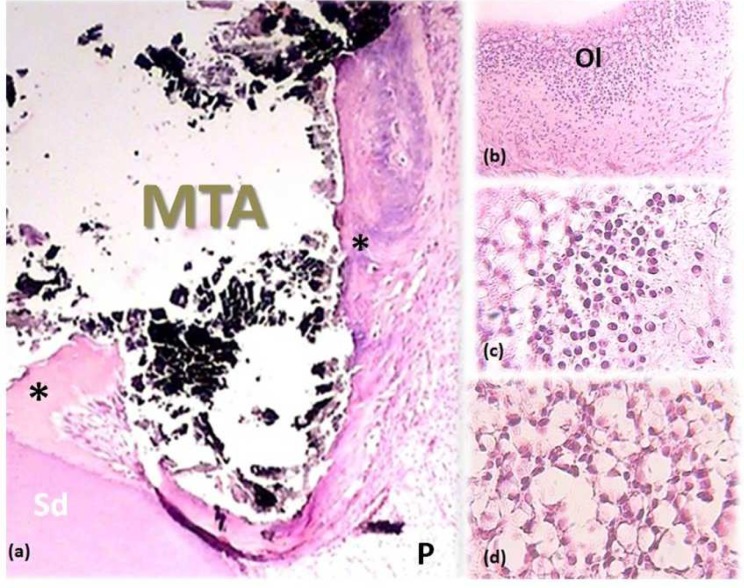
*A*) Complete calcified bridge formed (asterisks) after DPC with dexamethasone. Note the pulp (P) and secondary dentin (SD) (40×); *B*) An increase in cellular population in the odontoblastic layer (Ol) (100×); *C*) chronic inflammation; *D*) Odontoblast-like cells (400×)

**Figure 6:**
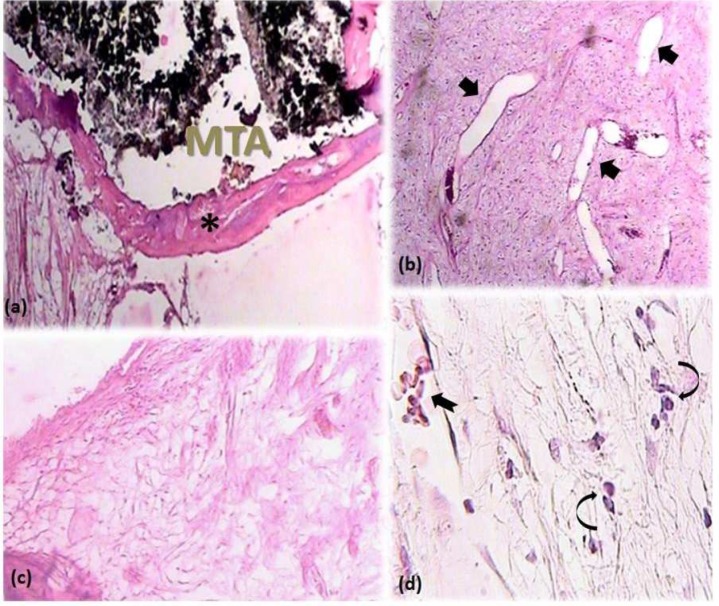
*A)* Complete calcified bridge formation (asterisk) after MP with dexamethasone (40×); *B*) Increased vascularity (arrows) of the pulp tissue (40×); *C*) Remarkable fibrous connective tissue formation and a decrease in pulp tissue cellularity (100×); *D*) Presence of chronic inflammatory cells (curved arrows) and multinucleated giant cells (notched arrow) represents pulp tissue reaction to the capping material (400×)

***Histological procedures***

Subsequent to extraction, apical one third of the tooth roots were immediately cut using high speed fissure burs and the teeth were immersed in a 10% neutral formalin solution (pH=7.4) for 2 weeks. They were decalcified in 10% nitric acid for 2 weeks. The decalcified specimens were washed in distilled water, dehydrated in an ascending ethanol series, and embedded in paraffin. Four-mµ thick buccolingual serial sections were prepared parallel to the tooth long axis and stained with Hematoxylin-eosin. Specimens were coded and submitted to the oral pathology department for histologic assessment of hard tissue barrier formation (pattern and continuity), foreign body reaction (FBR) to the capping material, type and status of inflammation, changes in collagen content and vasculature and granulation tissue formation (GTF). 

An experienced independent pathologist that was blind to the treatment procedure did the histologic evaluation by a light microscope (Leica BME, USA) linked to a digital camera (Axiocam ERc5s; Zeiss, G€ottingen, Germany) under 40, 100, and 400× magnifications. 

***Statistical analysis***

The data was statistically analyzed using the Kruskal-Wallis and Fisher Exact test. The SPSS software (SPSS version 9.0, SPSS, Chicago, IL, USA) served for calculating statistical means and statistical significance was set at 0.05.

## Results

None of the participants reported analgesic intake due to post-operative pain and discomfort. No radiographic signs of PDL widening or periapical pathosis were detected. Eventually, all specimens were attainable for microscopic assessment. GTF was not reported for any of the specimens. Calcified bridge formation was observed in all specimens (Figures 2 to 6). Pattern of calcified bridge was not significantly different between two treatment procedures (DPC and MP) (*P*>0.05). Topical dexamethasone did not have a significant effect on the pattern of calcified bridge formation (*P*>0.05). With regard to the continuity of the calcified bridge, MP showed more complete bridge formation than DPC regardless of dexamethasone application (Figure 3). However, the difference was not significant (*P*>0.05). 

FBR was observed in 4, 3, 3, and 2 specimens of DPC, MP, DPC+dexamethasone, and MP+dexamethasone, respectively. Although application of dexamethasone decreased the FBR following both DPC and MP procedures, the difference was not significant (*P*>0.05).

No acute inflammation was reported after sixty days. Severe inflammation was not evident in any of the specimens. Regarding to the type and severity of pulpal inflammation, there was no significant difference between DPC and MP (*P*>0.05). Type and severity of pulpal inflammation remained untouched by addition of dexamethasone to both DPC and MP procedures (*P*>0.05).

## Discussion

The aim of the current randomized clinical study was to compare the histologic pulp response to DPC and MP procedures using MTA with or without application of dexamethasone in healthy human premolars. Accordingly, the teeth were extracted and prepared for light microscopic examination after sixty days.

In this study, MTA was used as capping material because of its benefits including significant biocompatibility [23], sealing ability [24], regenerative capabilities [25], push-out bond strength properties [26] and antibacterial characteristics [27]. Nair *et al.* [28, 29] confirmed preliminary results of animal models in humans and emphasized that MTA should be considered the new gold standard for pulp-capping treatments [8]. Moreover, in a clinical trial by Banava *et al. *[9] which also compared the histologic human pulp response to DPC with MTA or calcium hydroxide, it was stated that much more favorable outcomes can be expected with MTA.

In the current study, one interesting finding was that there was no significant difference between MTA/DPC and MP concerning pulpal healing potential in mechanically exposed healthy human premolars. 

In the present study all teeth were symptom free throughout the study and calcified bridge was formed in all specimens providing more evidence for favorable outcome of one-step MTA-DPC and MP. The alkaline pH of MTA (10.2–12.5) seems responsible for the expression of the alkaline phosphatase, favoring the mineralization process in contact with organic tissues [30]. 

In case of pulpal exposures, inflammation is inevitable. Glucocorticoids block inflammatory mediator formation by inhibition of the arachidonic acid breakdown. In the current study, acute inflammatory reaction and severe inflammation were not reported for any of the specimens. Application of dexamethasone yielded more cases of “no inflammation” as well as less cases of “moderate inflammation” in both DPC and MP. However, the difference was not significant from statistical point of view. These findings are in line with previous studies showing limiting effect of corticosteroids on inflammatory reaction of dental pulp tissue [13, 15, 20, 31, 32] .

Some previous animal studies reported comparable hard tissue formation and reparative dentinogenesis to control groups following systemic corticosteroid treatments [15, 33]. The present study demonstrated that calcified bridge formation and pulp tissue healing were not hindered by topical dexamethasone application during DPC and MP. 

In human dental pulp cell culture, low concentrations of fluocinolone acetonide enhanced *type I* collagen synthesis [16]. In the present study, less increase in collagen content was observed following the application of dexamethasone. One possible explanation for this discrepancy is that the effect of glucocorticoids on collagen synthesis seems to be dose-dependent, and careful control of their release is of critical importance to successful pulp treatment [34].

Our results showed that vascular changes most probably remain untouched with topical dexamethasone application in DPC and MP. In a study, betamethasone application on the dentin reduced diameter and number of vessels in rat models [35]. Systemic damaging effects of corticosteroids seems highly unlikely in topical application during VPT procedures because of their small doses [36]. 

This study can be criticized in that intact teeth with no history of chronic inflammation were examined, so the pulp tissue reaction to VPT procedures was optimal and may not be the same in case of previously diseased pulp. However, this interferes to a lesser extent in case of mechanically iatrogenic exposures.

We recommend further studies using larger sample sizes to investigate the effects of corticosteroids on the outcome of VPT in cariously/traumatically exposed inflamed pulps.

## Conclusion

Within the limitations of this study, DPC and MP were histologically and clinically successful treatment options for VPT in mechanically exposed human teeth. Using proper technique, pulpal healing is anticipated with both one step DPC and MP procedures. For pulp healing capacity and outcome of VPT, proper technique exceeds topical application of corticosteroids.
